# Molecular biogeography and host relations of a parasitoid fly

**DOI:** 10.1002/ece3.5649

**Published:** 2019-09-26

**Authors:** David A. Gray, Henry D. Kunerth, Marlene Zuk, William H. Cade, Susan L. Balenger

**Affiliations:** ^1^ Department of Biology California State University Northridge Northridge CA USA; ^2^ Department of Ecology and Evolutionary Biology Cornell University Ithaca NY USA; ^3^ Department of Ecology, Evolution, and Behavior University of Minnesota St. Paul MN USA; ^4^ Department of Biological Sciences University of Lethbridge Lethbridge AB Canada; ^5^ Department of Biology University of Mississippi University MS USA

**Keywords:** *Gryllus*, host specialization, *Ormia*, parasitoid, range expansion, song distance matrix, *Teleogryllus*

## Abstract

Successful geographic range expansion by parasites and parasitoids may also require host range expansion. Thus, the evolutionary advantages of host specialization may trade off against the ability to exploit new host species encountered in new geographic regions. Here, we use molecular techniques and confirmed host records to examine biogeography, population divergence, and host flexibility of the parasitoid fly, *Ormia ochracea* (Bigot). Gravid females of this fly find their cricket hosts acoustically by eavesdropping on male cricket calling songs; these songs vary greatly among the known host species of crickets. Using both nuclear and mitochondrial genetic markers, we (a) describe the geographical distribution and subdivision of genetic variation in *O. ochracea* from across the continental United States, the Mexican states of Sonora and Oaxaca, and populations introduced to Hawaii; (b) demonstrate that the distribution of genetic variation among fly populations is consistent with a single widespread species with regional host specialization, rather than locally differentiated cryptic species; (c) identify the more‐probable source populations for the flies introduced to the Hawaiian islands; (d) examine genetic variation and substructure within Hawaii; (e) show that among‐population geographic, genetic, and host song distances are all correlated; and (f) discuss specialization and lability in host‐finding behavior in light of the diversity of cricket songs serving as host cues in different geographically separate populations.

## Graphical abstract

Geographic range expansion of a parasitoid fly has been associated with rapid exploitation of novel host species of crickets. Our genetic data detail that expansion and show that evolutionary specialization does not necessarily preclude continued flexibility.

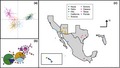

## 1 INTRODUCTION

Evolutionary specialization is often viewed as a double‐edged sword: Specialization may facilitate efficient exploitation of favored resources, but may also inhibit exploitation of novel resources. Specialization has often been viewed as an evolutionary “dead end” (Jaenike, 1990; Kelley & Farrell, 1998; Raia & Fortelius, 2013), although recent research has revealed considerable flexibility among specialist lineages and occasional “reversals” from specialized to more generalized niches (Gompert et al., 2015; Vamosi, Armbruster, & Renner, 2014). The retention of evolutionary lability may be especially relevant for geographic range expansion; indeed, “generalist” species are often among the most invasive (Romanuk et al., 2009)—a pattern found among plants, arthropods, mammals, and birds (Blackburn & Duncan, 2001; González‐Suárez, Bacher, & Jeschke, 2015; Higgins & Richardson, 2014; Snyder & Evans, 2006). For specialist species to expand their geographic range, they must readily encounter suitable resources, exhibit phenotypic plasticity enabling adoption of novel resources, and/or show rapid evolutionary adaptation.

Parasitoid insects, especially Ichneumonid and Braconid wasps (Hymenoptera) and Tachinid flies (Diptera), are especially illuminating for studies of host specialization, ranging from extreme generalists to extreme specialists (Quicke, 2014; Stireman, O'Hara, & Wood, 2006). Some species are sufficiently host‐specific to be used for classical biological control of pests (Parkman, Frank, Walker, & Schuster, 1996; Vargas, Leblanc, Putoa, & Eitam, 2007), others routinely utilize a broad range of hosts (Arnaud, 1978; Stireman, 2005; Tschorsnig, 2017), and in other cases, presumed generalists are later revealed to be complexes of cryptic specialists (Smith et al., 2008).

Within the ca. 9,000 species of Tachinids, the Ormiini tribe represents a small group (ca. 68 described species) of highly specialized flies (Lehmann, 2003; Sabrosky, 1953a, 1953b). Several specializations are noteworthy for the entire group (so far as is known): All are parasitoids of crickets or katydids (Ensifera, Orthoptera); all locate their (principally male) hosts using a specialized ear (Edgecomb, Robert, Read, & Hoy, 1995; Hedwig & Robert, 2014) to eavesdrop on their male host's mating song (Allen, 1995; Cade, 1975; Lehmann, 2003); and all have sclerotized planidiform larvae which are somewhat mobile and actively burrow into the host (Adamo, Robert, Perez, & Hoy, 1995; Cantrell, 1988). Within this group, all genera with known hosts parasitize katydids (Tettigoniidae); in the genus *Ormia*, most species parasitize katydids but three species attack crickets and mole crickets (Gryllidae and Gryllotalpidae; Lehmann, 2003). The shift from katydids to crickets and mole crickets represents a significant shift in female fly hearing toward lower frequency sounds (ca. 4–5 kHz in crickets and ca. 2–3 kHz in mole crickets) than are typical of most katydids (often >> 10 kHz). Utilization of katydids with relatively low frequency calls may have facilitated the evolutionary transition to crickets and mole crickets. For example, certain katydid hosts of Ormiines have relatively low frequency calls, for example, ca. 5–6 kHz in *Sciarasaga quadrata* (host of *Homotrixa alleni*; Allen, Kamien, Berry, Byrne, & Hunt, 1999); ca. 7 kHz in *Neoconocephalus robustus* (host of *O. brevicornis*; Nutting, 1953); and ca. 8 kHz in *Orchelimum pulchellum* (one of several hosts of *O. lineifrons*; Shapiro, 1995).

Within *Ormia*, *O. ochracea* has been most extensively studied. Peak sensitivity of female fly hearing closely matches or is at slightly higher frequencies than typical male host calling song (Robert, Amoroso, & Hoy, 1992). The current geographic range attributed to this species extends from Florida (Walker & Wineriter, 1991), across the southern Gulf States (Henne & Johnson, 2001), into Texas (Cade, 1975), Arizona (Sakaguchi & Gray, 2011), California (Wagner, 1996), and Mexico (Sabrosky, 1953b); throughout this range, it parasitizes various species of *Gryllus* field crickets (see below). In addition, *O. ochracea* was introduced to Hawaii by at least 1989 (Evenhuis, 2003), where it parasitizes *Teleogryllus oceanicus*, itself introduced to Hawaii from Australia via Oceania by at least 1877 (Kevan, 1990) and possibly earlier, perhaps facilitated by Polynesian settlement (Tinghitella, Zuk, Beveridge, & Simmons, 2011). Localized populations of *O. ochracea* show varying degrees of host specialization: Flies in Florida almost exclusively parasitize *Gryllus rubens* (Walker, 1993; Walker & Wineriter, 1991); flies in Texas primarily parasitize *G. texensis* (Cade, 1975); flies in Arizona regularly parasitize multiple *Gryllus* species (Sakaguchi & Gray, 2011); flies in southern California primarily parasitize *G. lineaticeps* (Wagner, 1996; Wagner & Basolo, 2007); and as noted above, Hawaiian flies parasitize *T. oceanicus*. Remarkably, playback experiments in Florida, Texas, California, and Hawaii, which simultaneously presented the songs of *G. rubens*, *G. texensis*, *G. lineaticeps*, and *T. oceanicus*, revealed that each fly population showed a significant (but not exclusive) preference for the song of its primary local host species of cricket (Gray, Banuelos, Walker, Cade, & Zuk, 2007). This suggests an even further degree of host specialization in these flies—possibly indicative of cryptic host races or species as has been found in other Tachinids (Smith et al., 2008; Smith, Woodley, Janzen, Hallwachs, & Hebert, 2006). Determining the extent to which geographic and host range subdivision is coupled with genetic subdivision is thus one of the goals of this study.

Successful establishment of *O. ochracea* in Hawaii represents a significant expansion of both the geographic and host range of the fly. How can such a specialist invade switch to a novel host with a strongly divergent song structure, and in the course of a few decades come to prefer that novel host's song to the songs of ancestral hosts? Two of our aims in this paper are to use mitochondrial and nuclear markers both to examine genetic variation within Hawaii and to identify the more‐likely continental source population(s) of those Hawaiian flies, and thereby the most likely types of recent ancestral host songs. This necessitates broad sampling of continental populations, and we therefore expand upon the previous work in the United States and include flies from populations in both northern and southern Mexico, as well as catalog the confirmed host species and their songs in each of these areas. We apply standard phylogeographic analyses to mitochondrial DNA sequence data, including outgroup species of *Ormia*, and we adopt a population genetic approach to analysis of microsatellite nuclear markers.

## 2 METHODS

### 2.1 Fly collection

We collected flies at mesh screen and/or bottle traps using playbacks of cricket songs (Walker, 1989). The songs played to attract flies varied among populations and across years, but for mainland sites always included songs of 2–4 species of crickets at least one of which was a known local host; for Hawaiian sites, some collections (WHC, 2003) were made with playbacks of four cricket songs (see Gray et al., 2007), whereas later collections used *T. oceanicus* song (the only Hawaiian host). We also collected a small number of flies at lights or as they emerged from field‐collected crickets. Table 1 provides details of locations and dates of sampling. Collected flies were preserved in ethanol until DNA extraction and further analysis. We extracted DNA using a Qiagen DNeasy tissue kit according to the manufacturer's instructions. We used entire flies as source tissue for all of the mainland and 13 of the Hawaiian flies, and head and thorax tissue for the remainder of the Hawaiian flies. In theory, the whole tissue extractions could include DNA from larvae, although the amounts of such DNA would be trivial compared to maternal DNA. We quantified DNA using a NanoDrop system and adjusted concentrations to between 20 and 75 ng/μl.

**Table 1:** Sample collection data; not all specimens were used in all analyses

Region	Locality	Dates	*N*	Collector(s)
Florida	Gainesville, FL	August 2002	41	DAG
Texas	San Antonio, TX	September 2002	5	WHC
	Austin, TX	September 2002, 2004	29	WHC and S. Walker 2002; DAG 2004
	Huntsville, TX	September 2002	1	S. Walker
Arizona	Sedona, AZ	August 2004	12	DAG
	Oak Creek, AZ	August 2004	6	DAG
	Holbrook, AZ	August 2002	1	DAG
	Verde River, AZ	August 2004	3	DAG
	Madera Canyon, AZ	August 2004	10	DAG
	KOFA, AZ	September 2005	2	DAG
	Yuma, AZ	November 2003	2	A. Izzo
	Parker Canyon, AZ	August 2004	2	DAG
	Petroglyph, AZ	September 2006	16	DAG
	Pinery Canyon, AZ	September 2004	5	DAG
	Portal, AZ	August 2003	1	DAG
Sonora	Alamos, Sonora, MX	July 2006	17	DAG
Oaxaca	San Pablo Etla, Oaxaca, MX	November 2014	13	DAG
California	Malibu Creek, CA	September and October 2003, 2004	22	DAG
	Stunt Ranch, CA	September 2002	10	DAG
	Santa Margarita Reserve, CA	September 2003	5	DAG
Hawaii	Kauai, HI	February and August 2014	24	MZ and SLB
	Hilo, HI	March 2003; February and August 2014	33	WHC 2003; MZ and SLB 2014
	Oahu, HI	February 2014	4	MZ and SLB
Outgroups				
*Ormia depleta*	Gainesville, FL	December 2003	2	H. Frank, via T. J. Walker
*Ormia lineifrons*	Gainesville, FL	December 2003	2	H. Frank, via T. J. Walker

### 2.2 Genetic markers and analysis

We analyzed population structure using both mitochondrial and nuclear markers. For mtDNA, we analyzed a section of *Cytochrome C Oxidase subunit I* (hereafter COI) PCR amplified in two overlapping fragments with “universal” primer pairs Jerry‐Pat and Ron‐Nancy (Simon et al., 1994), resulting in 1,111 bp after alignment. In addition, we developed nuclear microsatellite markers de novo for this project. Marker discovery was performed by 454 sequencing at the Cornell University Life Sciences Core Laboratories Center with further validation done by SLB and HDK. We identified and tested 17 msat markers from this dataset consisting of 3, 4, and 6 bp repeats. PCR conditions followed a “touchdown” protocol of 95° for 40 s, 66° for 45 s, and 72° for 45 s. The annealing step was reduced by one degree every cycle for the first seven cycles. Cycles 8–35 followed a pattern of 95° for 40 s, 58° for 45 s, and 72° for 45 s. PCR products were stored at −20°C until genotyped. Individuals were genotyped at microsatellite loci by the University of Minnesota Genomics Center on an Applied Biosystems 3730xl DNA Analyzer. We scored alleles for fragment size manually using Peak Scanner 2.0 software. Multiple independent analysts scored the same products to assure veracity of the calls. If no clear designation could be made or alleles did not amplify, we scored the data as missing.

### 2.3 Population genetics analyses

Prior to analysis of microsatellite fragments, we filtered individuals and loci for missing data. A strict cutoff of >25% missing data led to the exclusion of six loci. Following this filter, we excluded any individuals with missing data at three or more loci, resulting in the removal of 52 samples. The final dataset included 274 individuals genotyped at 11 loci with between 6 and 17 alleles per locus (Table 2); analyses were repeated after exclusion of three loci (see below). To estimate allelic richness and the number of private alleles accurately given unequal sample sizes per population, we performed a rarefaction analysis using HP‐Rare (Kalinowski, 2005) using the population with the smallest sample size (Oaxaca, 13 samples) to calculate adjusted values.

**Table 2:** Locus primer and allelic richness statistics

Locus	Primer sequence 5′‐3′	Repeat locus					Mean number of alleles								
		Motif	No.	Size (bp)	Pool	Dye	HI(K)	HI(O)	HI(H)	CA	AZ	SON	OAX	TX	FL
*Oo002*	F: GTGTGTGAGCGTCTGATCTTCC	CAGC	11	191	A	VIC	2.65	3.57	3.17 *	3.64	4.76	4.22	4.02	4.49 *	5.58
	R: ATCAGCCACATTTACACTTTCCC														
*Oo007*	F: TTCCTTTACTATCGTATTGGCGC	TTG	8	286	A	6‐FAM	1.99	2.41	2.20	5.27	5.46	5.11	6.73	4.69 *	4.68 *
	R: AGGAAGGAAGACAAACAAACAGC														
*Oo011*	F: CTGCCCTTTCACTCTTACTTGAC	AACGAC	14	395	A	PET	3.89	3.33	3.39 *	4.77	5.33	5.68	4.05	7.29	7.02
	R: GAGCTCCCTTGGCAAGTTAAATG														
*Oo017*	F: TCAAATATGGGCTGGTTTGGATG	TGGA	10	164	A	6‐FAM	2.00	2.00	1.99 *	3.36	4.97	5.49	6.44	5.05 *	6.01
	R: TGTCATGATGCAGCATAAACAAC														
*Oo022*	F: AAAGGTGTTAGAAGATGTTGGCG	GGAT	9	348	B	6‐FAM	3.61	2.56	2.58 †	6.29 *	7.97	6.51 *	8.40	7.73 *	7.34 †
	R: GATAATAGCGCTCGTAGTTGCAG														
*Oo024*	F: TATGACGTGCAGCAATATGAGTG	TTG	15	164	B	PET	2.54	2.24	2.22 †	2.89 †	3.77 *	3.93 *	3.52	3.48 *	3.69 *
	R: GTGACGTACGTTTGAAATGCTC														
*Oo028*	F: TCTTGTGGGTAATGGCAATTGTG	TAG	12	333	B	NED	2.00	2.41	2.18 *	4.69	5.97	7.04	6.68	5.30	5.76
	R: ATTTAATACGCAGCAATCCCAGG														
*Oo031*	F: ACATATGGTGAGTAGTGGATCCC	AAC	11	387	B	VIC	2.70	2.43	2.31 *	4.14	5.16 *	5.25	6.54	5.77	6.91
	R: ACCAGAAGCTGTCATATAGGGAG														
*Oo032*	F: TGAAGTGTGACAGTTTCTTGACG	TTG	12	416	A	VIC	2.94	3.21	3.36	5.79 *	5.86	4.47	6.28	7.09	6.34
	R: ACTGTCAAGGATGTTAAACTGGC														
*Oo034*	F: TTCGACCAAACCCATTATGTGAC	ACA	12	182	A	NED	1.92	1.83	1.90	1.90	2.78	3.02	3.59	3.34	3.25
	R: TCCGGACTATCGAGATTGTACTG														
*Oo035*	F: ATTTGCGGTGTTACTTCATTTGC	GTT	10	190	A	PET	1.33	2.06	1.43 *	2.64 *	4.72 *	6.14	6.08 *	6.28 *	6.98 †
	R: TTGCTTACCACTGTTCGCTAATC														
						Mean	2.51	2.55	2.43	4.12	5.16	5.17	5.67	5.50	5.78
						*SD*	0.73	0.55	0.60	1.32	1.25	1.13	1.53	1.42	1.32

Locus	Primer sequence 5′‐3′	Mean number of private alleles								
		HI(K)	HI(O)	HI(H)	CA	AZ	SON	OAX	TX	FL
*Oo002*	F: GTGTGTGAGCGTCTGATCTTCC	0.00	0.01	0.02	0.14	0.68	0.43	1.16	0.25	0.66
	R: ATCAGCCACATTTACACTTTCCC									
*Oo007*	F: TTCCTTTACTATCGTATTGGCGC	0.00	0.13	0.20	1.30	0.61	0.93	1.68	0.50	0.56
	R: AGGAAGGAAGACAAACAAACAGC									
*Oo011*	F: CTGCCCTTTCACTCTTACTTGAC	0.00	0.00	0.00	0.07	0.68	0.82	0.00	1.20	1.40
	R: GAGCTCCCTTGGCAAGTTAAATG									
*Oo017*	F: TCAAATATGGGCTGGTTTGGATG	0.00	0.00	0.00	0.00	0.02	0.51	1.18	0.37	1.05
	R: TGTCATGATGCAGCATAAACAAC									
*Oo022*	F: AAAGGTGTTAGAAGATGTTGGCG	0.00	0.00	0.00	0.82	1.18	0.84	1.42	1.46	1.54
	R: GATAATAGCGCTCGTAGTTGCAG									
*Oo024*	F: TATGACGTGCAGCAATATGAGTG	0.55	0.21	0.22	0.00	0.17	0.01	0.00	0.30	0.00
	R: GTGACGTACGTTTGAAATGCTC									
*Oo028*	F: TCTTGTGGGTAATGGCAATTGTG	0.00	0.41	0.20	0.64	0.58	0.16	0.39	0.18	0.21
	R: ATTTAATACGCAGCAATCCCAGG									
*Oo031*	F: ACATATGGTGAGTAGTGGATCCC	0.00	0.00	0.00	0.00	0.48	0.41	0.28	0.22	1.21
	R: ACCAGAAGCTGTCATATAGGGAG									
*Oo032*	F: TGAAGTGTGACAGTTTCTTGACG	0.00	0.24	0.38	1.23	0.92	0.14	1.23	1.26	0.86
	R: ACTGTCAAGGATGTTAAACTGGC									
*Oo034*	F: TTCGACCAAACCCATTATGTGAC	0.00	0.41	0.03	0.03	0.84	0.68	1.63	0.70	0.90
	R: TCCGGACTATCGAGATTGTACTG									
*Oo035*	F: ATTTGCGGTGTTACTTCATTTGC	0.00	0.41	0.00	0.07	0.63	0.79	0.34	1.15	1.87
	R: TTGCTTACCACTGTTCGCTAATC									
		0.05	0.17	0.10	0.39	0.62	0.52	0.85	0.69	0.93
		0.16	0.17	0.13	0.49	0.31	0.30	0.62	0.46	0.54

Note

Loci that significantly deviate from Hardy–Weinberg expectations in a population are marked with an *(if *p* < .05) or an ^†^(if *p* < .001) for that population; the three Hawaiian islands were pooled for HWE testing.

We visualized population genetic variation using a discriminant function analysis of principal components (DAPC) with 80 principal components and four discriminant functions using the *adegenet* (Jombart, 2008; Jombart & Ahmed, 2011) and *pegas* (Paradis, 2010) packages in R.

To visualize genetic structure, we implemented the Bayesian analysis program STRUCTURE v2.3.4 using an admixture model with correlated allele frequencies and without using source population as a prior. We used a burn‐in of 50,000 steps and 100,000 MCMC iterations. We conducted separate runs for the full dataset, a mainland dataset with the Hawaiian samples excluded, and a dataset of Hawaiian samples only. For the 8‐locus dataset, we performed 20 runs each for *k* = 1–9; for the 11‐locus dataset, we performed five runs each for *k* = 2–9. To infer the likely number of genetic clusters, we used both the Ln estimated probability of the data from STRUCTURE and the Evanno method utilizing Δ*k* (Evanno, Regnaut, & Goudet, 2005).

We calculated pairwise estimates of *F*_ST_ (Weir & Cockerham, 1984) and Nei's genetic distance between populations using the R packages *adegenet* and *ade4* (Chessel, Dufour, & Thioulouse, 2004), and we calculated expected and observed heterozygosity using *adegenet*. We tested if loci met Hardy–Weinberg expectations within each population (Hawaiian islands pooled) using an exact permutation test (Table 2).

To test for bottlenecks during a potential range expansion, we calculated the Garza and Williamson's *M* (Garza & Williamson, 2001) statistic for each population, with the three Hawaiian islands grouped as a single population.

We built a mitochondrial haplotype network using 55 haplotypes from 1,111 bp of COI sequences from 275 individuals using the R package *pegas* (Paradis, 2010) with default parameters.

### 2.4 Host ranges and songs

To provide context for understanding the degree of host specialization, we present in this paper the songs of confirmed hosts in each of the geographic regions studied. We present only hosts confirmed to be naturally parasitized by the development of *O. ochracea* from field‐collected crickets. We suspect that a few additional host species will be confirmed in the United States, especially if the species is only occasionally parasitized, and we expect that many more species are parasitized in southern and central Mexico; this reflects the status of current knowledge of *Gryllus* systematics and the extent of field sampling. Many of the confirmed host species are not yet officially described (Weissman and Gray, in press); to provide continuity within the literature, we use provisional manuscript names here and note that the names are disclaimed as unavailable per Article 8.3 of the ICZN.

Songs from field caught males were recorded in the laboratory, directly to computer at 44.1 kHz 16 bit sampling. In early work, we used CoolEdit 2000 and later switched to using Audacity (various versions over several years, currently v. 2.2.1). In an attempt to quantify relative song differences, we created a Euclidean song distance matrix using *matrix <‐ dist(songdata)* function in R. Song variables were dominant frequency (kHz), pulse rate, pulses per chirp or trill (ln‐transformed), pulse duty cycle, song type (chirp, trill, stutter‐trill, complex stutter‐trill), and chirps per trill (for stutter‐trillers), as well as introductory pulses per trill and introductory pulse rate (for complex stutter‐trillers). Prior to matrix calculation, the raw song data were normalized as *z*‐scores (see data and matrix in the accompanying data deposited in Dryad). The resulting song distance matrix has the advantage of objectively showing unit‐less quantitative differences among cricket host species, but has the disadvantage that the different song features are not weighted by their perceptual importance to *O. ochracea*, which would be preferable but is not currently possible. Our coding of song characters is only one of many possible coding schemes; our goal was to capture the major structural differences among cricket songs (Alexander, 1962) while attempting to have song features coded in such a way that comparisons across species represent “homologous” traits in song space, see Desutter‐Grandcolas and Robillard (2003).

We used Mantel tests implemented in the R package *ecodist* (Goslee & Urban, 2007) to relate the cricket host song distances among the different fly populations to the geographic and genetic distances among the fly populations. Geographic distances were measured from Google Earth as terrestrial linear distances (i.e., avoiding crossing the Gulf of Mexico). We used Nei's genetic distances, both including and excluding three loci identified as deviating from Hardy–Weinberg expectations in five or more populations (see Section 3). We used the among‐cricket‐species song distance matrix (described above) to generate an among‐fly‐populations song distance matrix. This is a complicated endeavor because several fly populations regularly utilize multiple cricket hosts, so the song differences among the fly populations represent the song differences among an assemblage of cricket host species, not between the songs of single species of crickets. We could not settle on an a priori “best” way to do this, so we tried three approaches: (a) pairwise average song distance between fly populations for commonly utilized host species, (b) pairwise minimum song distance between fly populations for commonly utilized host species, and (c) pairwise minimum song distance between fly populations for all known host species. Method (i), the average song distance between the commonly utilized hosts conceptually represents how different are the suite of host cues for the host species most relevant to the evolutionary fitness of the flies. Method (ii), the minimum song distance between the commonly utilized hosts conceptually represents the minimum difference in recognition of host cues necessary for the fly to establish a population within a new geographic area with a particular assemblage of potential hosts and have high fitness with at least one host. Method (iii), minimum song distance for all known hosts conceptually represents the minimum difference in recognition of host cues necessary for the fly to have any fitness within a new assemblage of potential host species. The “commonly utilized host species” per population was determined based on prior fieldwork (Gray et al., 2007; Hedrick & Kortet, 2006; Sakaguchi & Gray, 2011; Walker & Wineriter, 1991; Weissman & Gray, in press) and was as follows: Florida *G. rubens*; Texas *G. texensis*; Arizona *G. “longicercus*,*” G. “staccato*,*” G. “regularis*,*” G. armatus*, and *G. cohni*; Sonora *G. “staccato*,*” G. “regularis*,*” G. armatus*, and *G. cohni*; Oaxaca *G. assimilis*; California *G. lineaticeps* and *G. integer*; and Hawaii *T. oceanicus*.

## 3 RESULTS

### 3.1 Nuclear and mitochondrial genetics

Three loci (*Oo022*, *Oo024*, and *Oo035*) showed significant departure from Hardy–Weinberg expectations in five or more populations (Table 2); subsequent analyses were done both including and excluding these three loci. Following filtration at missing data cutoffs, 274 individuals and either 11 or 8 loci (see above) were included in the final msat dataset, with 1.86% data missing. Heterozygosity across all individuals was 50.9% (11 loci) or 56.0% (8 loci). The Hawaiian populations showed a drastic decrease in heterozygosity (Table 3). The rarefaction analysis also suggested a substantial decrease in both total and private allelic diversity within the Hawaiian populations (Table 2).

**Table 3:** Population sample sizes and heterozygosity for nuclear msat loci

Population	Sample size	No. alleles	Heterozygosity (expected)	Heterozygosity (observed)
Kauai	20	29 (21)	0.437 (0.480)	0.367 (0.460)
Oahu	28	31 (22)	0.438 (0.479)	0.367 (0.450)
Hilo	32	34 (26)	0.401 (0.449)	0.321 (0.400)
California	32	62 (47)	0.588 (0.591)	0.478 (0.528)
Arizona	57	95 (71)	0.667 (0.665)	0.612 (0.625)
Sonora	17	70 (52)	0.677 (0.658)	0.588 (0.648)
Oaxaca	13	70 (53)	0.724 (0.723)	0.607 (0.604)
Texas	35	91 (67)	0.714 (0.709)	0.604 (0.636)
Florida	40	95 (70)	0.741 (0.730)	0.638 (0.693)

Note

Values are given for the full dataset of 11 loci with values in parentheses for the reduced set of eight loci.

**Figure 1:**
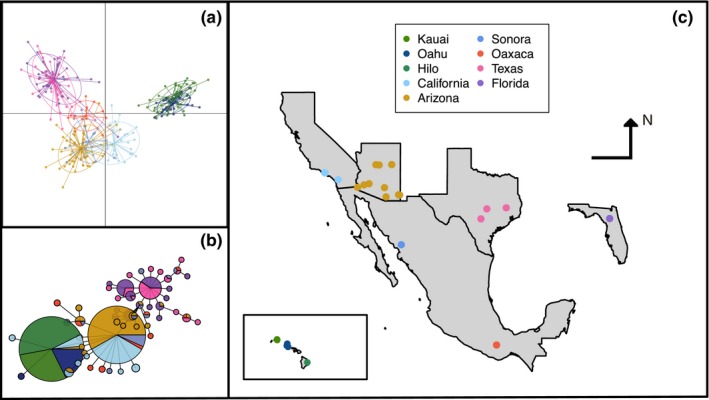
(a) DAPC clustering analysis. Individuals are marked as points with ellipses representing 75% of the observed data. (b) Haplotype network of 55 haplotypes of 1,111 bp of mitochondrial COI gene sequences. (c) Map of collection sites

Analysis of Nei's genetic distances documented a clear split between Hawaiian and mainland populations (Table 4), with Hawaiian populations more similar to western mainland populations. Longitude explained the primary axis of variation among the mainland populations, with a clear east–west gradient evident in both the DAPC and mtDNA haplotype network (Figure 1). Pairwise comparisons of Fst demonstrated a similar east–west pattern for mainland populations, but did not show a clear pattern between Hawaiian and mainland populations (Table 4).

**Table 4:** Pairwise *F*_ST_ (above diagonal) and Nei's genetic distance (below diagonal) by population

	Kauai	Oahu	Hilo	California	Arizona	Sonora	Oaxaca	Texas	Florida
Kauai		0.027 (0.029)	0.057 (0.066)	0.092 (0.102)	0.071 (0.080)	0.105 (0.099)	0.109 (0.108)	0.091 (0.086)	0.087 (0.083)
Oahu	0.044 (0.033)	–	0.047 (0.056)	0.088 (0.089)	0.079 (0.084)	0.099 (0.087)	0.095 (0.090)	0.100 (0.093)	0.098 (0.091)
Hilo	0.096 (0.063)	0.073 (0.055)	–	0.114 (0.115)	0.097 (0.100)	0.124 (0.110)	0.118 (0.111)	0.127 (0.117)	0.122 (0.113)
California	0.263 (0.327)	0.229 (0.299)	0.279 (0.310)	–	0.024 (0.028)	0.034 (0.030)	0.049 (0.056)	0.055 (0.059)	0.060 (0.065)
Arizona	0.282 (0.395)	0.267 (0.378)	0.291 (0.369)	0.088 (0.132)	–	0.011 (0.008)	0.019 (0.023)	0.031 (0.035)	0.035 (0.038)
Sonora	0.290 (0.365)	0.286 (0.359)	0.344 (0.375)	0.127 (0.163)	0.067 (0.089)	–	0.032 (0.037)	0.026 (0.028)	0.026 (0.031)
Oaxaca	0.327 (0.443)	0.305 (0.451)	0.365 (0.456)	0.235 (0.293)	0.151 (0.197)	0.169 (0.189)	–	0.022 (0.027)	0.021 (0.024)
Texas	0.331 (0.427)	0.332 (0.450)	0.394 (0.454)	0.231 (0.263)	0.165 (0.171)	0.149 (0.167)	0.158 (0.205)	–	0.008 (0.006)
Florida	0.337 (0.442)	0.336 (0.451)	0.388 (0.461)	0.273 (0.303)	0.187 (0.190)	0.167 (0.202)	0.171 (0.211)	0.045 (0.058)	–

Note

Values are given for the full dataset of 11 loci with values in parentheses for the reduced set of eight loci.

**Figure 2:**
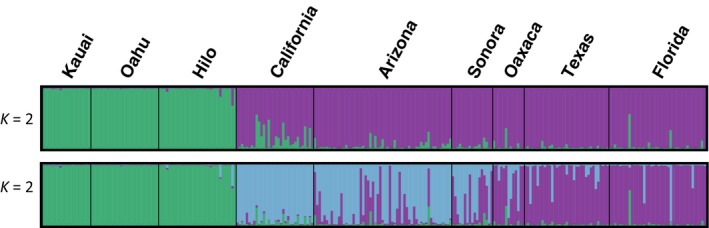
Bayesian clustering analysis implemented by STRUCTURE software (Pritchard, Stephens, & Donnelly, 2000). Top panel shows clustering into two genetic groups (*K* = 2), and the bottom panel shows clustering into three genetic groups (*K* = 3)

For the 8‐locus dataset, with all samples, STRUCTURE analyses indicated the strongest support for *k* = 2 genetic clusters (mean LnP(*K*) = −6286.49) separating Hawaiian from mainland populations (Figure 2); however, support for *k* = 3 clusters was also high (mean Ln*P*(*K*) = −6028.0), which further divided the mainland populations into eastern and western subsets (Figure 2). The Evanno method indicated the strongest support for *k* = 2 clusters (Table S1). STRUCTURE plots for within Hawaii (*k* = 2 and *k* = 3) and mainland (*k* = 2, *k* = 3, and *k* = 6) are in Figures S1 and S2. Analysis of the 11‐locus dataset (5 runs, *k* = 2–9) gave qualitatively the same results: strongest support for *K* = 2 genetic clusters (mean Ln*P*(*K*) = −8386.5, Delta *K* = 131.43), though support for *K* = 3 clusters was also high (mean Ln*P*(*K*) = −8082.08, Delta *K* = 68.13).

**Figure 3:**
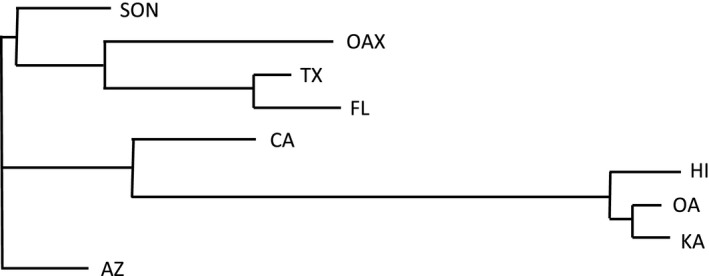
Unrooted neighbor‐joining of populations based on multilocus microsatellite genetic distances (Nei's distances, 8 loci)

The mtDNA haplotype network (Figure 1b) also showed (a) low genetic variation within Hawaii, (b) affinity of the Hawaiian sequences for the western mainland (i.e., California) sequences, and (c) a longitudinal geographic structure within the mainland populations. Oaxaca had a high diversity of haplotypes shared with all other mainland populations. Neighbor‐joining analysis of populations based on Nei's genetic distance (eight loci) also shows affinity of California and Hawaii (Figure 3).

**Figure 4:**
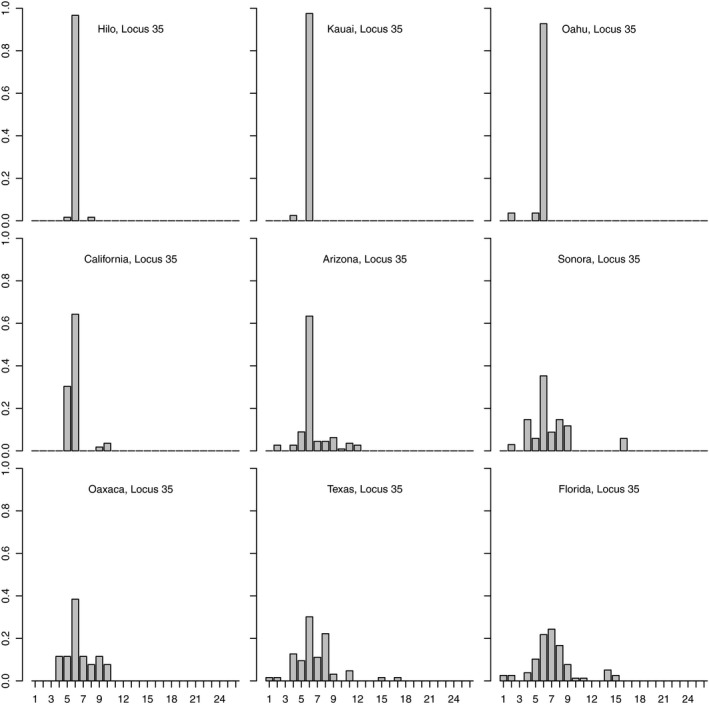
Allele frequency histograms for msat locus 35 for each population

**Figure 5:**
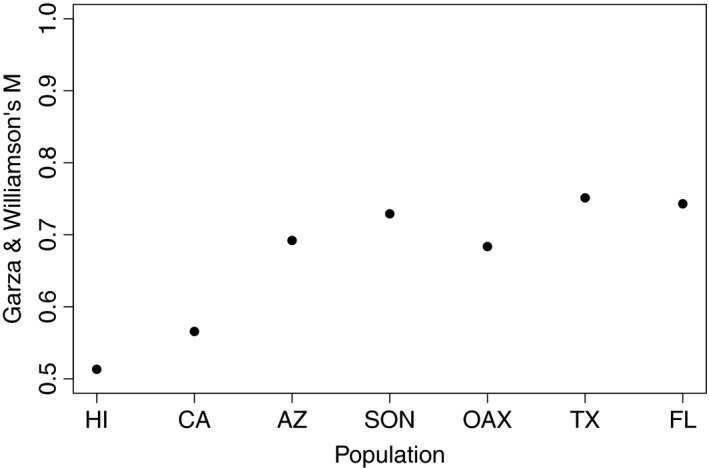
Garza and Williamson's M for each population, suggesting genetic bottlenecks associated with reductions in population size, especially for California and Hawaii

Given the apparent distinctness of the Hawaiian populations, it is important to emphasize that these patterns reflect founder effects, and concomitant change in allele frequency in Hawaii, not the development of novel genetic variation in Hawaii. This is most easily seen in allele frequency histograms which show that the Hawaiian genetic variation is effectively a simple subset of the genetic variation found in western mainland populations, themselves a simple subset of the genetic variation found in Florida, Texas, and Mexico populations (see Figure 4 for a representative locus; figures for all other loci show similar patterns and are presented as Figures S3–S12). The Garza and Williamson's *M* statistic also provided support for bottlenecks due to founder effects in the Hawaiian populations (Figure 5) and a more modest reduction in population size as the range expanded westward (e.g., California).

### 3.2 Host range and song structures

**Figure 6:**
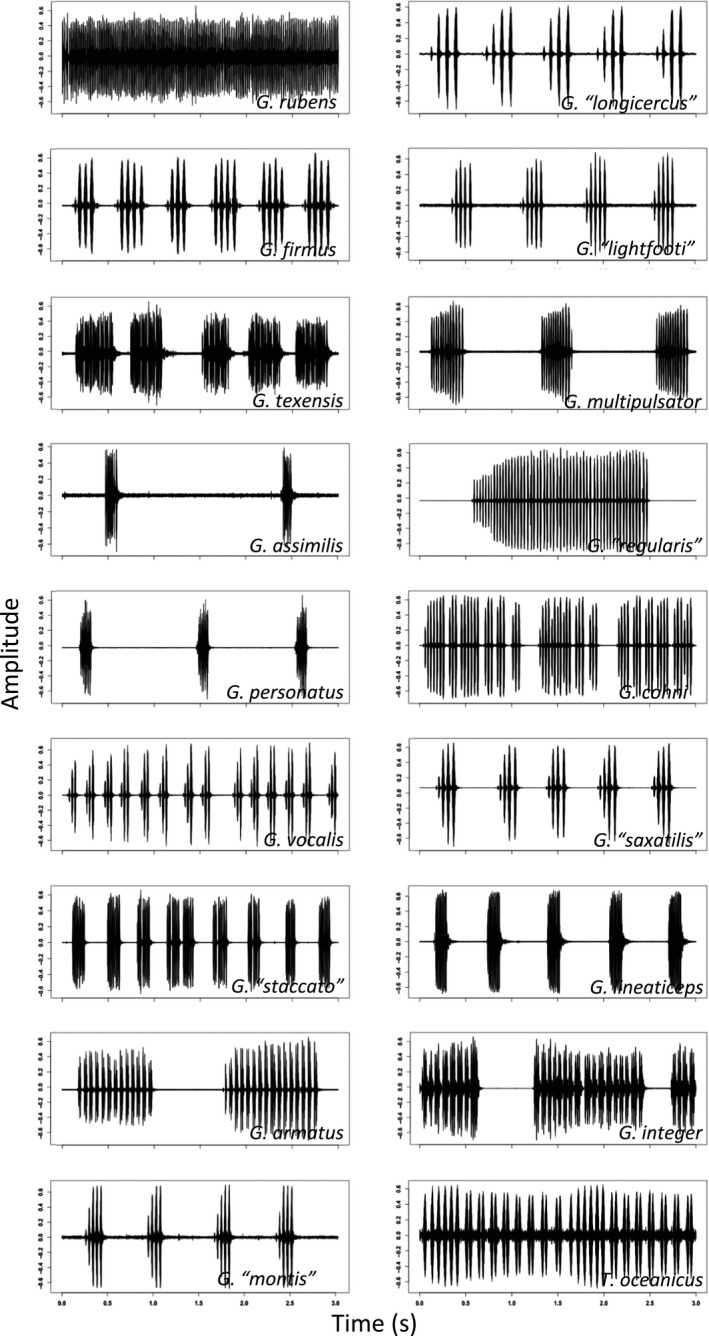
Waveform oscillograms of 3 s of song from confirmed host species showing overall song structure (chirps/trills)

**Figure 7:**
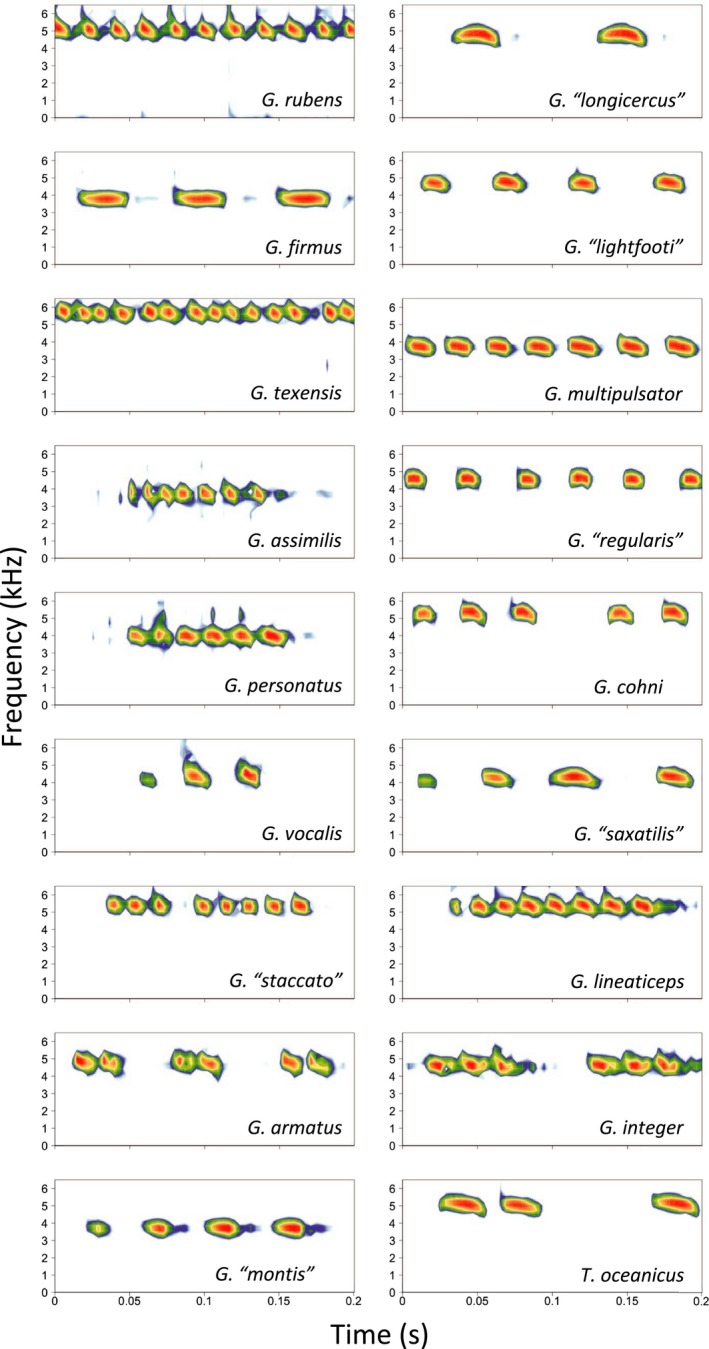
Spectrogram representations of 0.2 s of song from confirmed host species showing fine‐scale song structure (pulses)

Confirmed host species, geographic range information, and host calling song type, frequency, pulse rate, and pulses/chirp are presented in Table 5. Songs of confirmed host species vary dramatically, from simple chirps to complex trills; see waveform oscillograms and frequency spectrograms in Figures 6 and 7, respectively (prepared using the R package *seewave*).

**Table 5:** Confirmed hosts of *Ormia ochracea*

Host species	Confirmed as host in	Song type	Dominant frequency (kHz)	Pulse rate (p/s)^a^	Pulses per chirp or trill^b^	References for host status and song data
*G. rubens*	Florida	Trill	4.7	50–55	100–200	Blankers, Hennig, and Gray (2015), Izzo and Gray (2004), Vélez and Brockmann (2006), and Walker and Wineriter (1991)
*G. firmus*	Florida, Texas	Chirp	4.2	16	3–5	Doherty and Storz (1992), Walker and Wineriter (1991), and D. Weissman personal communication
*G. texensis*	Texas, Oklahoma, Coahuila	Trill	5.2	75–80	25–65	Blankers et al. (2015), Cade (1975, 1981), Cade, Ciceran, and Murray (1996), Gray and Cade (1999), Izzo and Gray (2004), DAG, and D. Weissman personal communication
*G. assimilis*	Texas, Oaxaca, Nuevo Leon	Chirp	3.7	85	6–9	DAG; D. Weissman personal communication (Weissman, Walker, & Gray, 2009)
*G. personatus*	Arizona, Coahuila	Chirp	4.0	57	6–8	DAG; D. Weissman personal communication (Gray, Gutierrez, et al., 2016)
*G. vocalis* a.k.a. Regular stutter‐triller	Arizona	Fast chirp	4.8	33	3–4	D. Weissman personal communication (Sakaguchi & Gray, 2011; Weissman, Rentz, Alexander, & Loher, 1980)
*G. “staccato”* a.k.a. G#15	Arizona, Sonora	Chirp	5.2	73	6–8	Gray, Gutierrez, et al. (2016), Sakaguchi and Gray (2011), and DAG
*G. armatus*	Arizona	Stutter‐trill	3.6	58	2, 15–20	Hedrick and Kortet (2006), and DAG
*G. “montis”*	Arizona	Chirp	3.8	22	4–5	DAG
*G. “longicercus”* a.k.a. G#13	Arizona	Chirp	4.5	10	4–6	DAG; D. Weissman personal communication (Gray, Gabel, Blankers, & Hennig, 2016)
*G. “lightfooti”*	Arizona	Chirp	4.5	20	4–6	DAG; D. Weissman personal communication
*G. multipulsator*	Arizona, Sonora, Jalisco, Zacatecas, Sinaloa, Baja California Sur	Chirp	4.1	70	12–16	A. Izzo; DAG; D. Weissman personal communication (Weissman et al., 2009)
*G. “regularis”* a.k.a. G#14, Arizona triller	Arizona	Trill	4.5	38	20–80	Blankers et al. (2015), Sakaguchi and Gray (2011), and DAG
*G. cohni* a.k.a. G#20, Arizona stutter‐triller	Arizona, Sonora	Stutter‐trill	4.8	25	2–8, 1–6	Sakaguchi and Gray (2011), and DAG
*G. “saxatilis”* a.k.a. G#2	California, Baja California Norte	Chirp	4.1	20	3–4	DAG; D. Weissman personal communication
*G. lineaticeps*	California	Chirp	5.1	55	6–8	Gray, Gutierrez, et al. (2016), Wagner (1996), Wagner and Basolo (2007), and DAG
*G. integer*	California	Stutter‐trill	4.5	60	2–3, 15–80	Hedrick and Kortet (2006), Hedrick and Weber (1998), Paur and Gray (2011a) and Weissman et al. (1980)
*Teleogryllus oceanicus*	Hawaii	Complex 2‐part trill// stutter‐trill^c^	4.6	14//24	6−8//2, 8–10	Zuk, Simmons, and Cupp (1993) and Zuk, Simmons, and Rotenberry (1995)

a Pulse rates approximate the average at 25°C.

b For stutter‐trillers, numbers are given as pulses per chirp, chirps per trill.

c For the *T. oceanicus* 2‐part song, numbers are given as trill part 1//stutter‐trill part 2.

**Figure 8:**
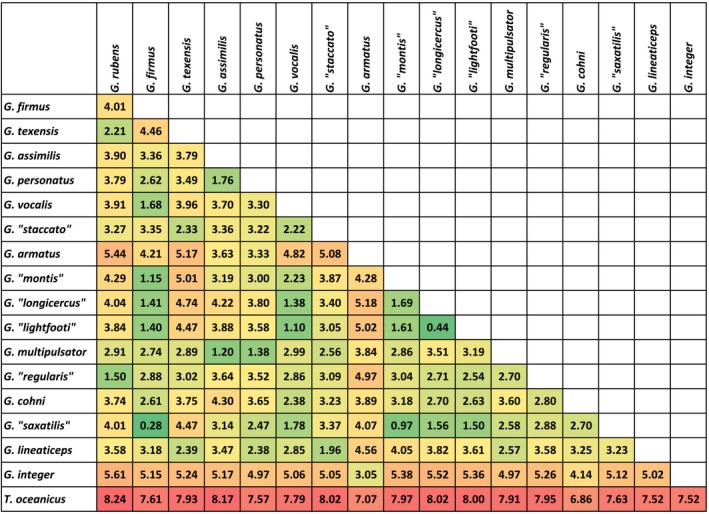
Euclidean pairwise interhost song distances with heatmap colors indicating similar songs (green) or strongly divergent songs (red)

The song distance matrix shows nearly 30‐fold variation in pairwise interhost song distance comparisons (0.28 between *G. “saxatilis”* and *G. firmus* vs. 8.24 between *G. rubens* and *T. oceanicus*; Figure 8). Notably, the average distance of *T. oceanicus* song from each of the other songs was about double the average distances for the continental *Gryllus* species (7.75 vs. 3.85, *Z* = 7.4, *p* < .0001).

**Figure 9:**
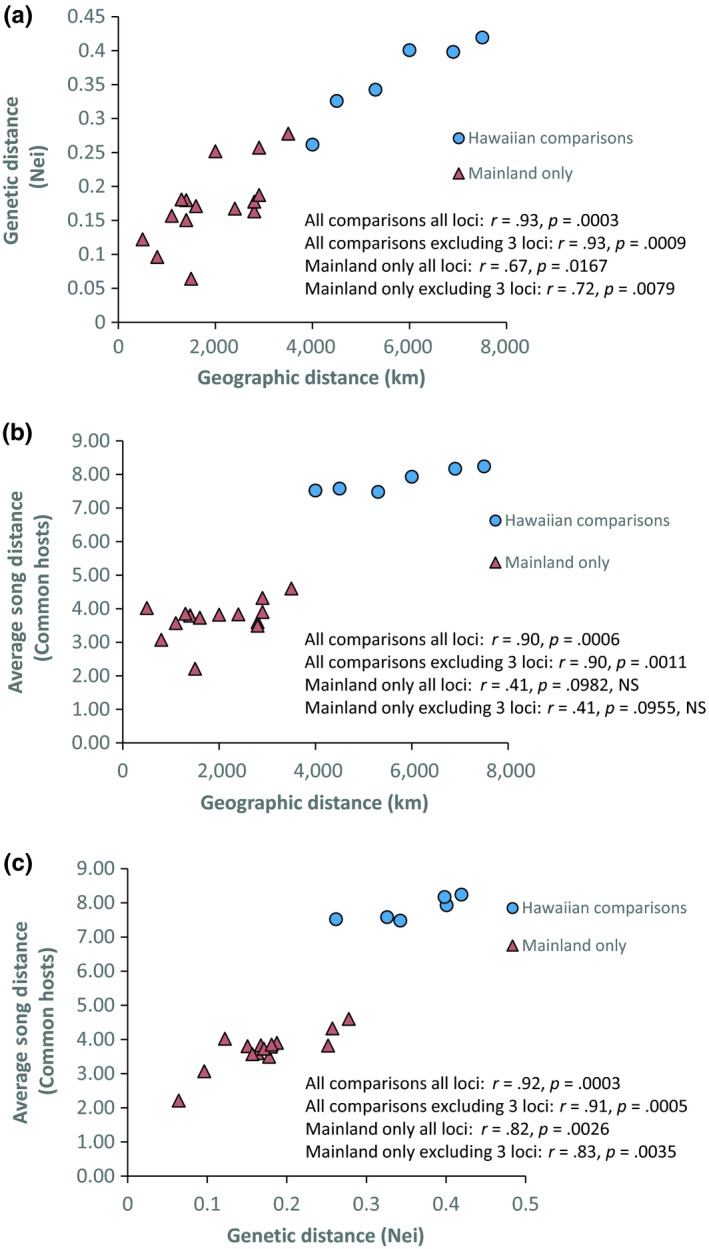
(a–c) Association between geographic, genetic, and song distances among populations

**Figure 10:**
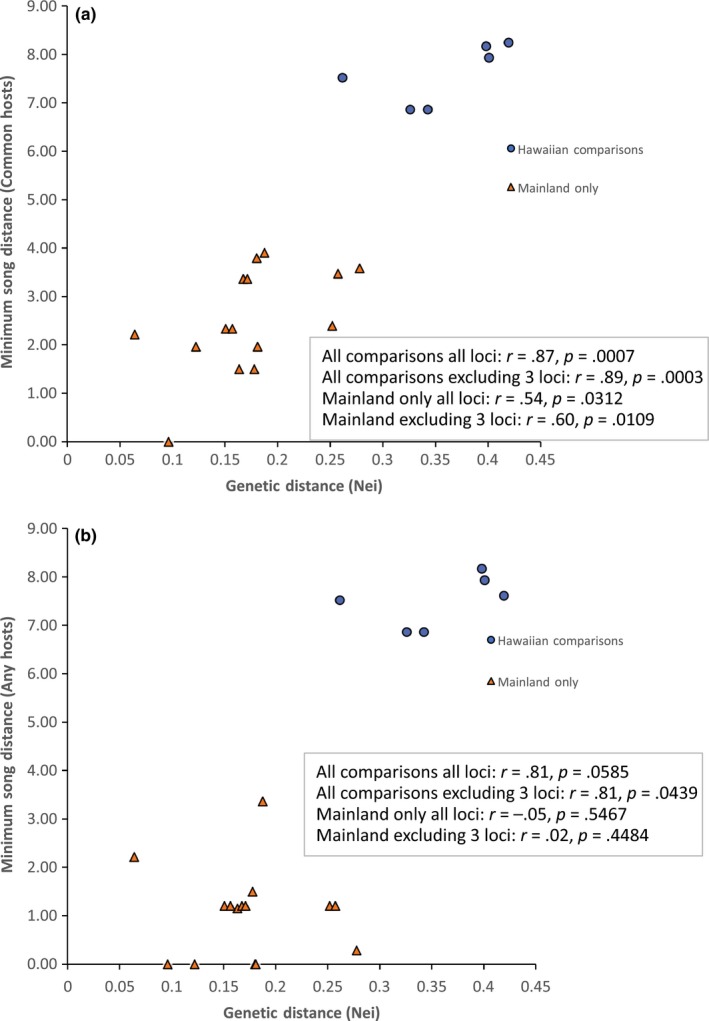
(a,b) Association between genetic and song distances among populations

Mantel tests showed strong associations between geographic, genetic, and song distances (Figures 9 and 10). To explore these patterns further, we repeated the analyses excluding the comparisons based on Hawaiian samples, that is, Mantel tests just for mainland population comparisons. Using average song distances among common hosts, song distance was correlated with genetic distance both when considering all comparisons and when considering only mainland comparisons (Figure 9c); the same was true when using minimum song distance among common hosts (Figure 10a), but not minimum song distance among any hosts (Figure 10b).

Partial Mantel tests gave somewhat inconsistent results (Table 6). Across all analyses, generally, it appears that the correlation between genetic and geographic distances persists even after conditioning on song distance. Song distance was significantly correlated with genetic distance, after conditioning on geographic distance, only for mainland comparisons using average song distance among commonly used hosts. The same pattern was not significant but somewhat suggested for all comparisons using average song distance among commonly used hosts, and for mainland comparisons using minimum song distance among commonly used hosts. Using minimum song distances among any hosts resulted in no relationship, or even a negative relationship, between song distance and genetic distance after conditioning on geographic distance.

**Table 6:** Results of partial Mantel tests of among‐population matrices of genetic (Nei), geographic (km), and host song distances

Population comparisons	Song distance method	M1 ~ M2 + M3	All loci		Excluding 3 loci	
			*r*	*p*	*r*	*p*
All comparisons	Average b/w common hosts	Gen ~ Geo + Song	.60	.0856	.61	.0812
		Geo ~ Song + Gen	.32	.2167	.34	.1856
		Gen ~ Song + Geo	.51	.0807	.48	.0866
	Minimum b/w common hosts	**Gen ~ Geo + Song**	**.67**	**.0084**	**.66**	**.0122**
		Geo ~ Song + Gen	.39	.1277	.37	.1305
		Gen ~ Song + Geo	.34	.1506	.38	.1101
	Minimum b/w any hosts	**Gen ~ Geo + Song**	**.78**	**.0004**	**.78**	**.0007**
		Geo ~ Song + Gen	.63	.0621	.63	.0648
		Gen ~ Song + Geo	−.09	.6518	−.10	.6562
Mainland only	Average b/w common hosts	**Gen ~ Geo + Song**	**.63**	**.0236**	**.75**	**.0049**
		Geo ~ Song + Gen	−.31	.8273	−.49	.9538
		**Gen ~ Song + Geo**	**.80**	**.0023**	**.84**	**.0001**
	Minimum b/w common hosts	**Gen ~ Geo + Song**	**.57**	**.0354**	**.64**	**.0311**
		Geo ~ Song + Gen	.13	.3544	.01	.4571
		Gen ~ Song + Geo	.37	.1632	.45	.0905
	Minimum b/w any hosts	**Gen ~ Geo + Song**	**.76**	**.0035**	**.79**	**.0013**
		**Geo ~ Song + Gen**	**.61**	**.0111**	**.59**	**.0193**
		Gen ~ Song + Geo	−.49	.982	−.46	.9032

Note

Comparisons in bold are significant at *p* < .05.

Abbreviations: Gen, genetic distance (Nei); Geo, geographic distance (km); M1, response matrix; M2, explanatory matrix; M3, conditional matrix; Song, song distance.

## 4 DISCUSSION

Our results suggest the following: (a) *O. ochracea* is a single widespread species with regional host specialization, not a complex of cryptic species, (b) *O. ochracea* has spread geographically into northern Mexico (Sonora) and the western United States (Arizona and California) from source populations in southern Mexico (Oaxaca) and/or the southern US Gulf region (Florida, Texas), (c) Hawaiian flies were introduced from a western continental US population, most likely California, potentially consisting of as few as one gravid female fly, and (d) novel song types with highly divergent song structures do not inhibit novel host exploitation. We elaborate on these results below and discuss mechanisms of regional host song specialization.

Studies of other Tachinid groups have sometimes revealed that what was considered a single generalist species actually consists of a complex of cryptic specialist species (Smith, Wood, Janzen, Hallwachs, & Hebert, 2007; Smith et al., 2006). The regional host specialization in *O. ochracea* documented previously (Gray et al., 2007) could have been consistent with either a widespread generalist with regional host preferences or with multiple cryptic host specialists. Both the mtDNA and msat variation suggest a single species. The mtDNA sequences, although showing clear east–west geographic structure, are relatively uniform and strongly divergent from *O. depleta* and *O. lineifrons* sequences (Figure S13). The msat data clearly show that populations strongly differentiated in host song preferences can nonetheless be genetically panmictic. Perhaps the best example of this involves flies from Florida and Texas: Gray et al. (2007) showed that Florida flies preferred *G. rubens* song over *G. texensis* song nearly 2:1 and that Texas flies preferred *G. texensis* song over *G. rubens* song 6:1. Nonetheless, the pairwise *F*_ST_ of 0.008 for these populations (Table 4) and the DAPC (Figure 1a) show that these two populations are genetically rather homogenous.

Both the mtDNA and msat data also inform the broader geographic history of the fly within North America. There is a clear east–west differentiation among samples, consistent with isolation by distance (Figure 9a). Moreover, the pattern of allelic variation in the msat loci (e.g., Figures 4 and S3–S12) suggests serial founder effects as flies colonized the western continental United States and then Hawaii; this interpretation is supported by Garza and Williamson's *M* (Figure 5). The mtDNA similarly suggests that the older fly lineages are to be found within the southeastern US populations (Figures 1b and S13). In this light, it is interesting to note that Florida is home to two *Gryllus* species, *G. ovisopis* and *G. cayensis*, which lack a normal calling song (Gray, Hormozi, Libby, & Cohen, 2018; Walker, 1974, 2001), possibly a consequence of a prolonged history of *Ormia* parasitism in that region. In contrast, there are no noncalling *Gryllus* in western North America. If the southeastern United States was an original source area for western North American populations, then the ancestral host songs were likely simple trills as in *G. rubens* and *G. texensis*.

The introduction of *O. ochracea* to Hawaii appears virtually certain to have been from a western North American population. The dominant mtDNA haplotype in Hawaii is also found in California and Arizona (Figure 1b); locus by locus, the msat allelic variation in Hawaii is likewise a subset of the most common alleles in California and Arizona (Figures 4 and S3–S12); combining msat loci, a neighbor‐joining tree based on Nei's distances places Hawaii and California as sister populations (Figure 3). A single introduction seems likely; the levels of genetic variation in Hawaii do not preclude the possibility that the introduction could have consisted of as few as one gravid female, although it seems more plausible that multiple individuals were introduced, perhaps as pupae in soil. In other systems, experimental introductions have indicated that in some circumstances, introductions of a single gravid female can nonetheless establish a persistent population (Fauvergue, Malausa, Giuge, & Courchamp, 2007; Grevstad, 1999). Within Hawaii, our data are consistent with the spread of an introduced population among islands, rather than separate introductions on each island (Figure S1).

Once in Hawaii, the adoption of *T. oceanicus* as a host represents a major shift within *O. ochracea's* repertoire of host song recognition. Quantitatively and qualitatively, *T. oceanicus* song is strikingly divergent from the songs of continental North American hosts (Figures 6, 7, 8). Even within mainland sites only, song divergence is associated with both geographic and genetic distances (Figures 9 and 10), demonstrating adoption of hosts with novel songs. Across the diversity of host songs, one could argue that the single essential song recognition feature is a dominant frequency in the 3–6 kHz range. This may be true in a strict sense, but frequency is clearly not the only song recognition feature. Multiple studies have shown that the temporal pattern of sound pulses is also important (Gray & Cade, 1999; Sakaguchi & Gray, 2011; Wagner, 1996; Wagner & Basolo, 2007; Walker, 1993). Moreover, fly populations prefer the temporal structure of their most common host species, even when dominant frequencies are similar (Gray et al., 2007). Perhaps most remarkably, Gray et al. (2007) showed that Hawaiian *O. ochracea* preferred *T. oceanicus* song over the songs of ancestral host species by a large margin (12 of 13 Hawaiian flies chose *T. oceanicus* song over the songs of *G. rubens*, *G. texensis*, and *G. lineaticeps*).

Adoption of *T. oceanicus* as a host in Hawaii also required compatible host physiology for larval development. Although mostly confined to parasitism of adult males, *O. ochracea* can develop within a wide variety of crickets, including juveniles (Vincent & Bertram, 2009) and species not normally used as hosts (Adamo, Robert, & Hoy, 1995; Thomson, Vincent, & Bertram, 2012) including *Acheta domesticus* (Paur & Gray, 2011a, 2011b; Wineriter & Walker, 1990) which is more distantly related to *Gryllus* than is *Teleogryllus* (D. A. Gray, D. B. Weissman, E. M. Lemmon, A. R. Lemmon, unpublished data). This latitude probably results from the generalized nature of the cricket immune encapsulation response (Vinson, 1990), which is exploited by Ormiines to develop a respiratory spiracle. Given this latitude, we expect that physiological compatibility with *T. oceanicus* was unlikely to be a significant factor in terms of host suitability.

Our results suggest that host specialization in *O. ochracea* is not at odds with rapid exploitation of novel hosts, as might be expected from evolutionary theory (Jaenike, 1990; Kelley & Farrell, 1998; Raia & Fortelius, 2013), despite associations between song divergence and genetic divergence independent of geography. But how can highly regional host song specificity (Gray et al., 2007), even to the point of flies having song preferences for certain intraspecific song variants (Gray & Cade, 1999; Sakaguchi & Gray, 2011; Wagner, 1996; Wagner & Basolo, 2007), be compatible with flexible and rapid adoption of novel hosts? We expect that behavioral plasticity coupled with local host learning (Paur & Gray, 2011a) may be the mechanism that enables flies to escape the “dead end” of specialization.

## CONFLICT OF INTEREST

None declared.

## AUTHOR CONTRIBUTIONS

DAG, SLB, MZ, and WHC conceived of the study and collected flies; DAG performed the mtDNA sequencing; SLB and HDK performed the msat amplification and analysis; and all authors contributed to the writing and editing of the manuscript.

## Data Availability

The COI sequence data have been deposited in GenBank with accession numbers MK522523–MK522797. Other supporting data are available in Dryad https://doi.org/10.5061/dryad.h8t823r.
