# Supplementary material for: Molecular biogeography and host relations of a parasitoid fly
Source: Ecol Evol. 2019 Sep 26;9(19):11476–93. doi: 10.1002/ece3.5649 (PMC6802024; doi:10.1002/ece3.5649)

Supplemental Materials

Gray et al. Molecular biogeography and host relations of a parasitoid fly.

Table S1. Composite STRUCTURE results for the 8 locus analysis with mean likelihoods and Delta K values calculated using the Evanno method.

|  | K | Reps | Mean LnP(K) | Stdev LnP(K) | Ln'(K) | \|Ln''(K)\| | Delta K |
| --- | --- | --- | --- | --- | --- | --- | --- |
| All Populations | 1 | 20 | -6949.08 | 0.0951 | NA | NA | NA |
|  | 2 | 20 | -6286.49 | 1.7097 | 662.59 | 404.1 | 236.358123 |
|  | 3 | 20 | -6028 | 2.0097 | 258.49 | 247.935 | 123.368346 |
|  | 4 | 20 | -6017.445 | 11.0271 | 10.555 | 29.13 | 2.641681 |
|  | 5 | 20 | -5977.76 | 6.1718 | 39.685 | 165.305 | 26.783979 |
|  | 6 | 20 | -6103.38 | 80.485 | -125.62 | 157.455 | 1.956328 |
|  | 7 | 20 | -6071.545 | 101.5047 | 31.835 | 155.410 | 1.531061 |
|  | 8 | 20 | -6195.12 | 57.32 | -123.575 | 12.765 | 0.222697 |
|  | 9 | 20 | -6331.46 | 76.8655 | -136.34 | NA | NA |
|  |  |  |  |  |  |  |  |
| Mainland Only | 1 | 20 | -5237.68 | 0.1436 | NA | NA | NA |
|  | 2 | 20 | -5003.82 | 15.7783 | 233.86 | 233.1 | 14.773436 |
|  | 3 | 20 | -5003.06 | 15.9533 | 0.76 | 30.425 | 1.907133 |
|  | 4 | 20 | -4971.875 | 10.4657 | 31.185 | 143.605 | 13.72152 |
|  | 5 | 20 | -5084.295 | 74.5334 | -112.42 | 169.665 | 2.276363 |
|  | 6 | 20 | -5027.05 | 75.3331 | 57.245 | NA | NA |
|  |  |  |  |  |  |  |  |
| Hawaii Only | 1 | 20 | -1028.405 | 0.1605 | NA | NA | NA |
|  | 2 | 20 | -1050.88 | 23.7555 | -22.475 | 5.615 | 0.236367 |
|  | 3 | 20 | -1067.74 | 13.4245 | -16.86 | 62.445 | 4.651569 |
|  | 4 | 20 | -1147.045 | 91.2554 | -79.305 | NA | NA |


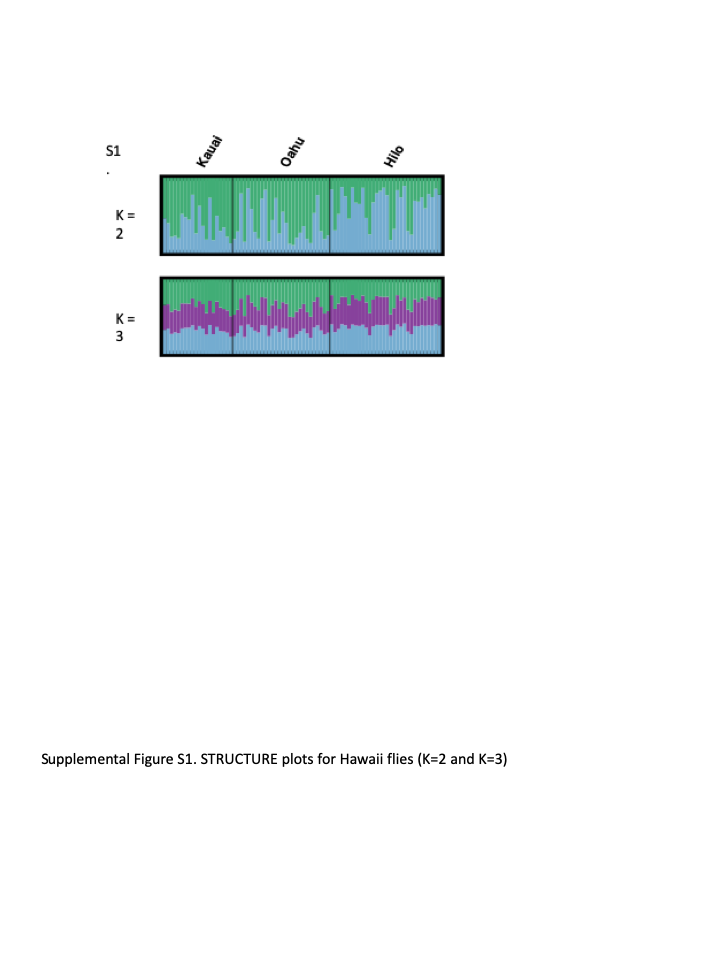


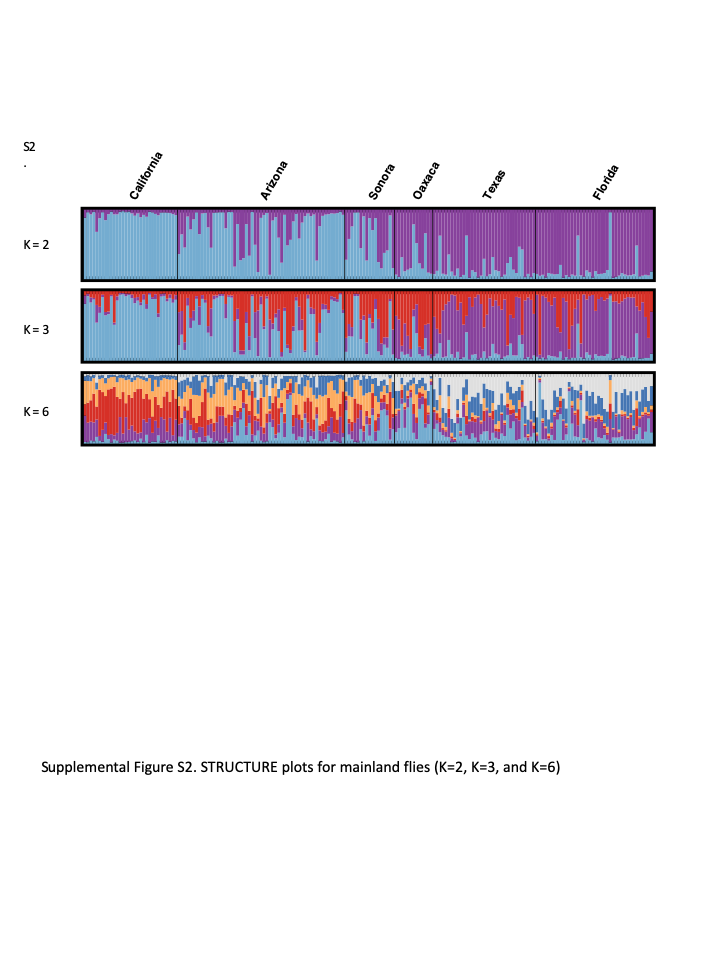


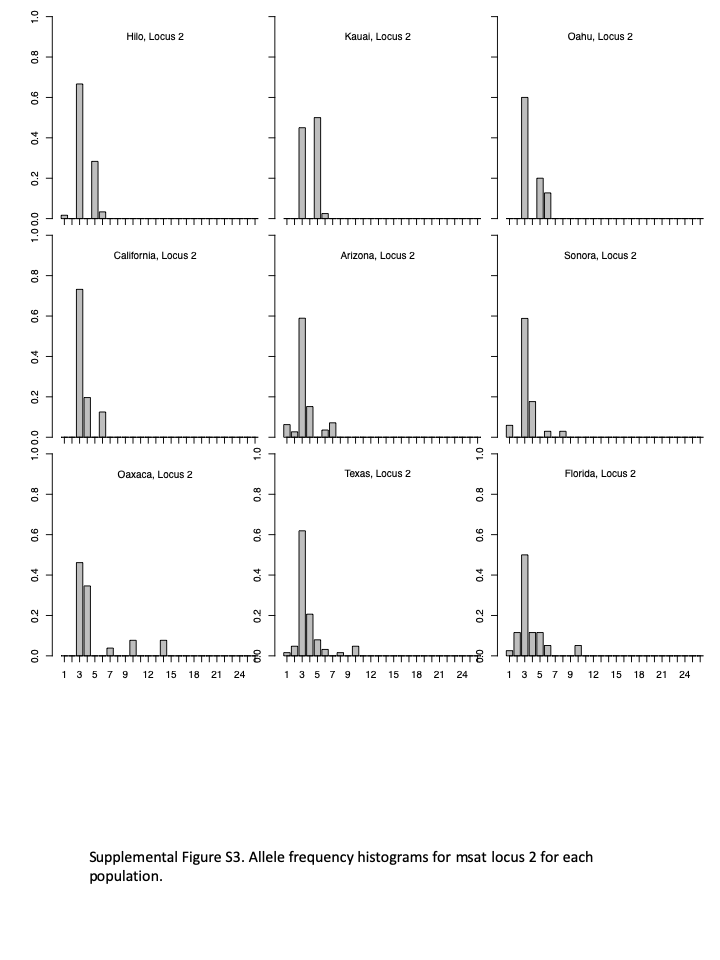


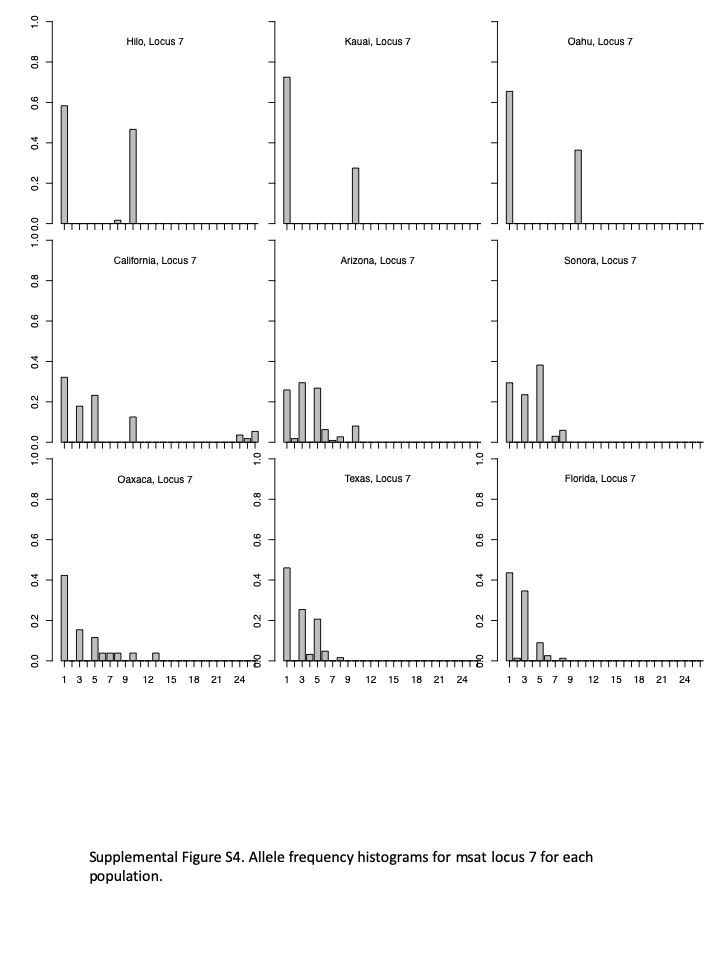


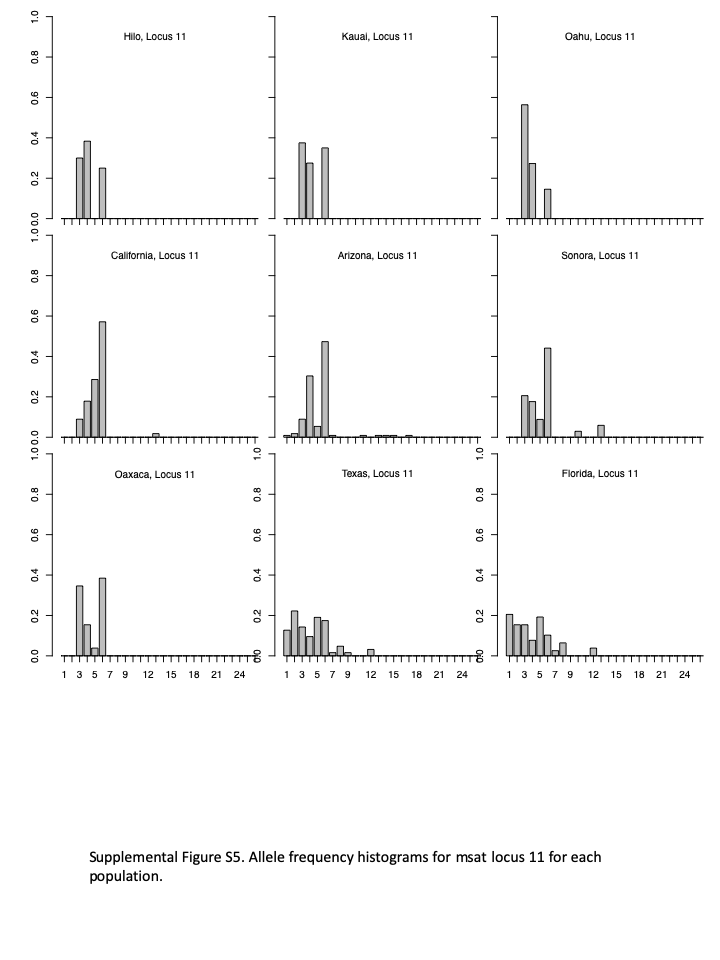


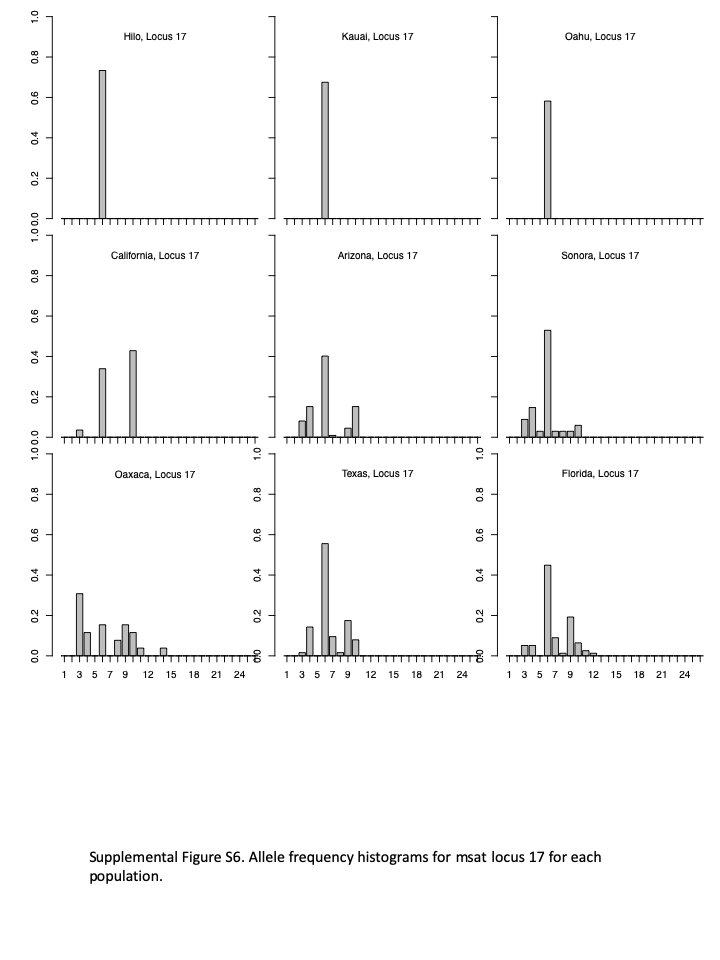


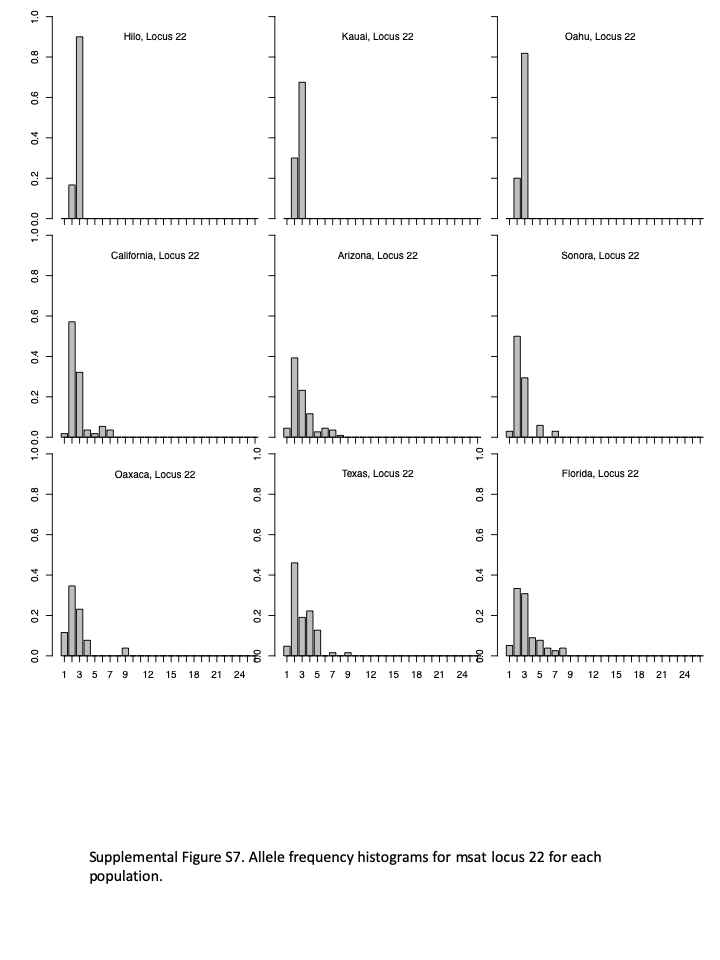


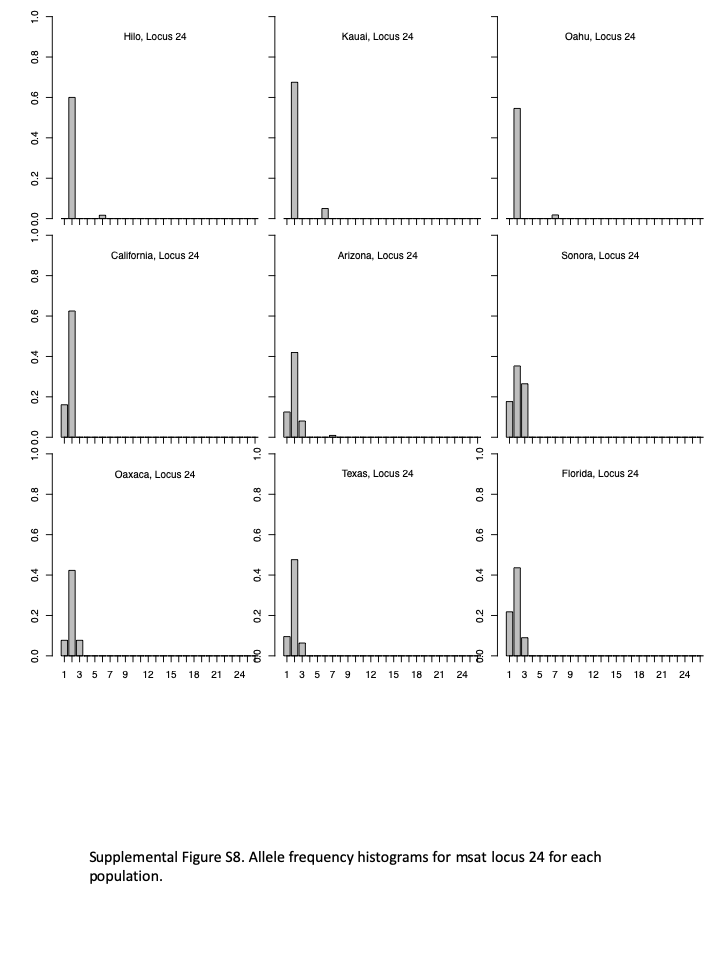


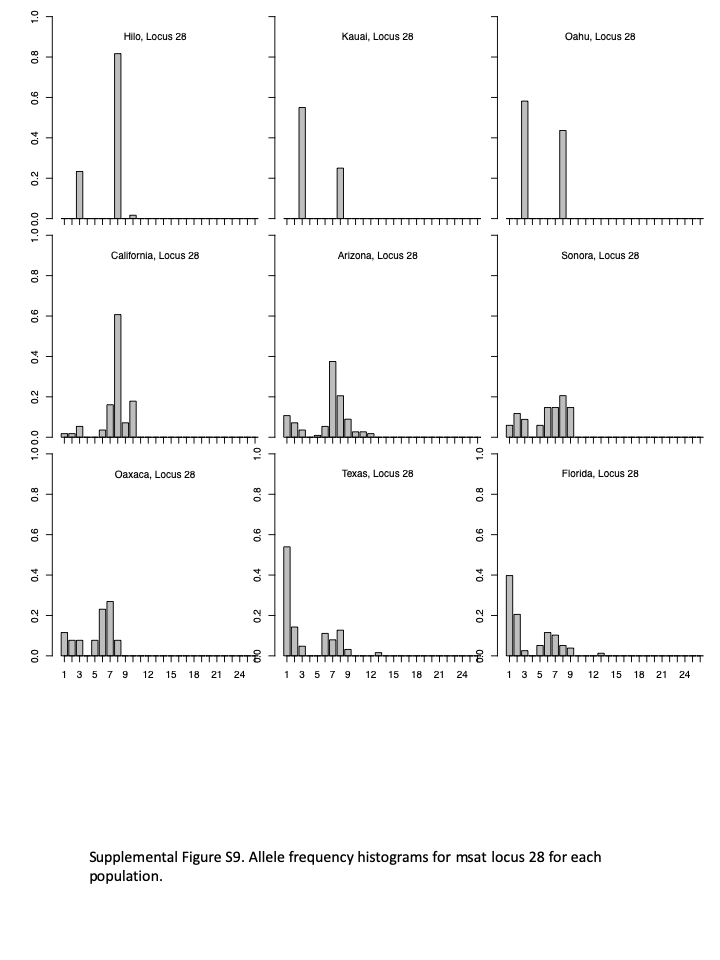


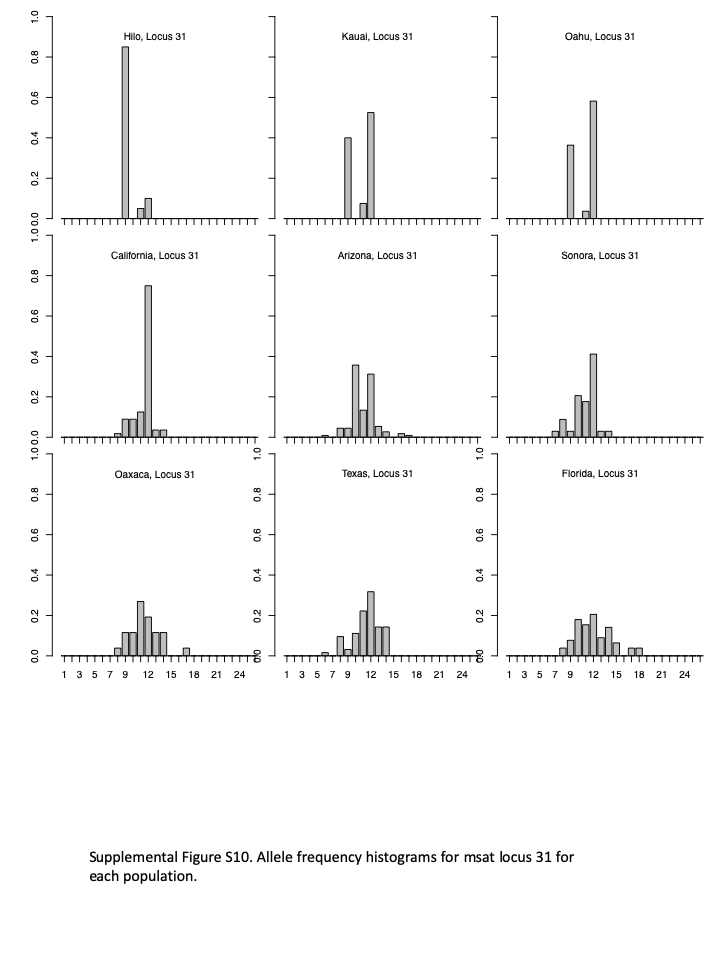


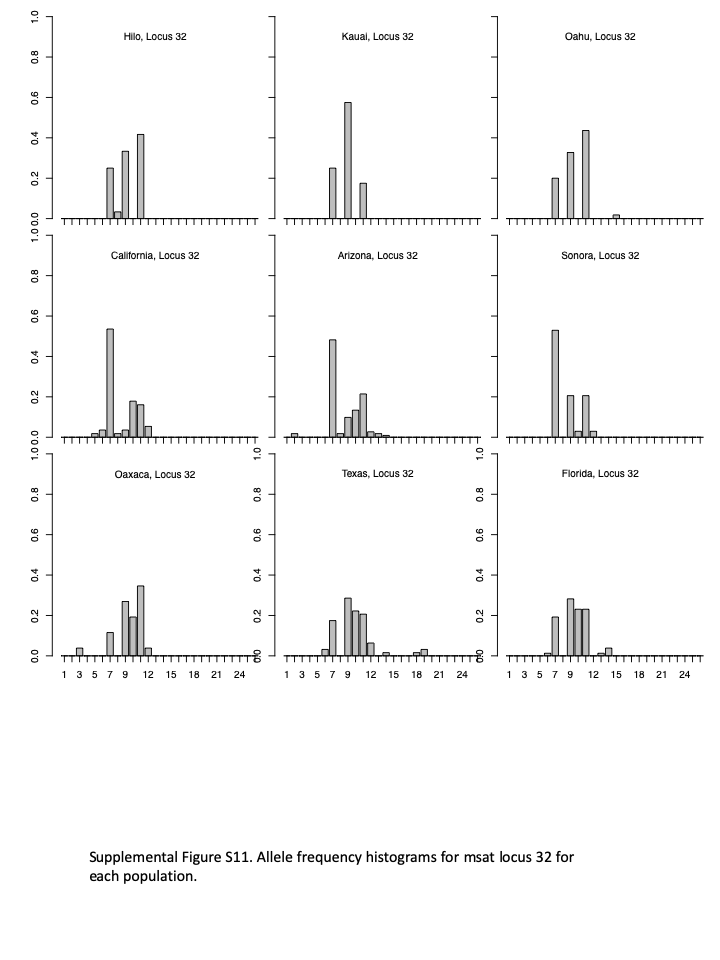


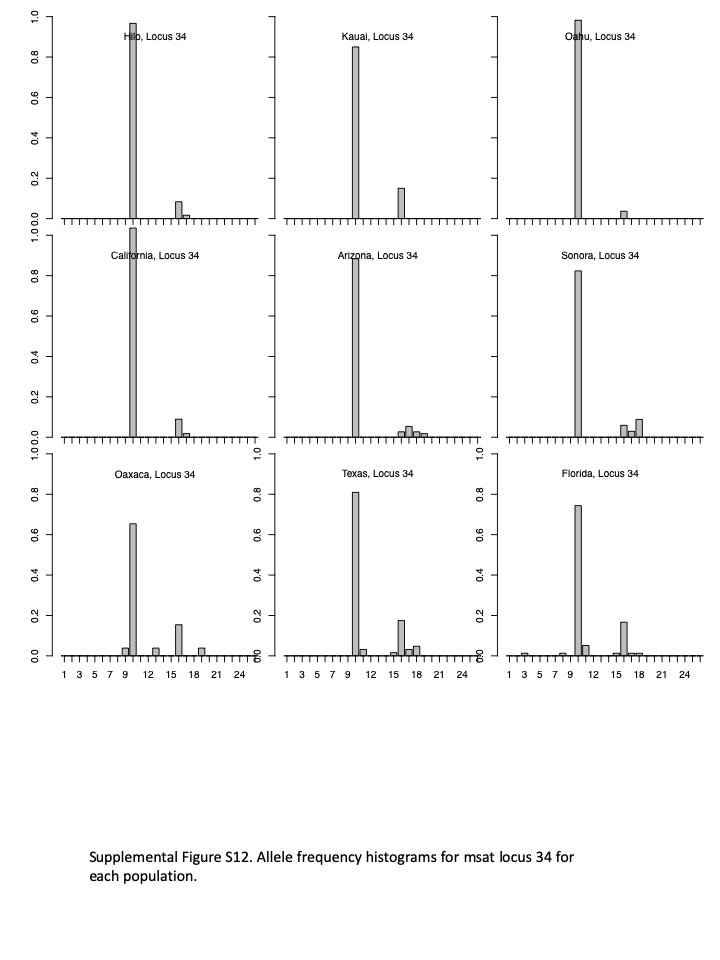


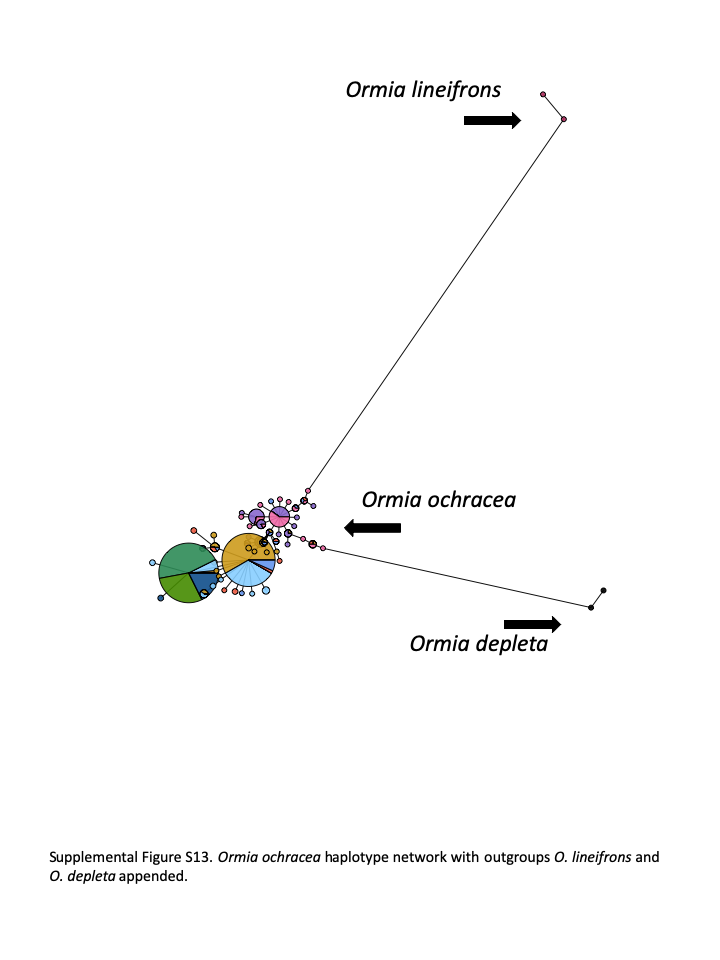

Supplement: Supplementary file 1 [file ECE3-9-11476-s001.docx]
